# In adults, early mobilization may be beneficial for distal radius fractures treated with open reduction and internal fixation: a systematic review and meta-analysis

**DOI:** 10.1186/s13018-021-02837-0

**Published:** 2021-11-24

**Authors:** ZhiBo Deng, JiangPing Wu, KaiYing Tang, Han Shu, Ting Wang, FuBing Li, Mao Nie

**Affiliations:** 1 Department of Orthopaedic Surgery, Second Affiliated Hospital of Chongqing Medical University, Chongqing, 400010 China; 2 Department of Orthopaedic Surgery, 920Th Hospital of Joint Logistics Support Force, Kunming, 650032 Yunnan Province China

**Keywords:** Meta-analysis, Distal radius fracture, Open reduction internal fixation, Early mobilization, Late mobilization

## Abstract

**Objectives:**

It remains debatable if early mobilization (EM) yields a better clinical outcome than the late mobilization (LM) in adults with an acute and displaced distal radial fracture (DRF) of open reduction internal fixation (ORIF). Therefore, we aimed to perform a systematic review and meta-analysis of randomized controlled trials (RCTs), comparing clinical results with the safety of EM with LM following ORIF.

**Methods:**

Databases such as Medline, Cochrane Central Register, and Embase were searched from Jan 1, 2000, to July 31, 2021, and RCTs comparing EM with LM for DRF with ORIF were included in the analysis. The primary outcome of study included disabilities of the Arm, Shoulder, and Hand (DASH) score at different follow-up times. Wherever the secondary outcomes included patient-rated wrist evaluation (PRWE), grip strength (GS), visual analog scale (VAS), wrist range of motion (WROM), and associated complications, the two independent reviewers did data extraction for the analysis. Effect sizes of outcome for each group were pooled using random-effects models; thereafter, the results were represented in the forest plots.

**Results:**

Nine RCTs with 293 EM and 303 LM participants were identified and included in the study. Our analysis showed that the DASH score of the EM group was significantly better than LM group at the six weeks postoperatively (− 10.15; 95% CI − 15.74 to − 4.57, *P* < 0.01). Besides, the EM group also had better outcomes in PRWE, GS and WROM at 6 weeks. However, EM showed potential higher rate for implant loosening and/or fracture re-displacement complication (3.00; 95% CI 1.02–8.83, *P* = 0.05).

**Conclusion:**

Functionally, at earlier stages, EM for patients with DRF of ORIF may have a beneficial effect than LM. The mean differences in the DASH score at 6 weeks surpassed the minimal clinically important difference; however, the potentially higher risk of implant loosening and/or fracture re-displacement cannot be ignored. Due to the lack of definitive evidence, multicenter and large sample RCTs are required for determining the optimal rehabilitation protocol for DRF with ORIF.

*PROSPERO registration number*: CRD42021240214 2021/2/28.

**Supplementary Information:**

The online version contains supplementary material available at 10.1186/s13018-021-02837-0.

## Introduction

Distal radius fracture (DRF) is one of the most common fracture [1, 2]. Particularly in an aging society, the incidence of DRF will continue to grow [3, 4]. Despite the existing variations, in the view of significantly better results in the reduction and functional recovery of open reduction internal fixation (ORIF) [5], it has become the primary surgical technique for the treatment of such fractures [6, 7]. However, rehabilitation type and immobilization duration following the plate fixation of DRFs remain uncertain [8], whereas persistent plaster fixation has been repeatedly questioned as a conventional rehabilitation program [9]. For patients in the early mobilization (EM) group, the satisfaction level remains higher due to the self-opportunity of maintaining basic hygiene without any protective measures [10]. However, EM is controversial due to the local pain-associated complications, poor wound healing, implant loosening, loss of reduction, and internal fixation failure [11].

A randomized controlled study demonstrated that EM positively impacted the surgical treatment outcome and caused no additional complications compared to late immobilization (LM) [12]. Furthermore, a prospective study revealed that EM had better patient-reported outcomes and wrist range of motion (WROM). Meanwhile, it did not require multiple follow-ups and guidance from physiotherapists during rehabilitation [13]. Another study reported that the LM does not lead to decreased wrist motion compared to initial wrist motion [10, 14]. Furthermore, Andrade et al., have reported the comparative more use of opioids in the early active groups [15]. In the light of these results, after the surgery, the postoperative fixation time ranges from immediate mobilization to the 6 weeks of cast immobilization, based on the different practices of the surgeons [13, 16–18].

To the best of our knowledge, no evidence-based medical study compared the EM with the conventional LM after ORIF of DRF. Therefore, we performed systematic review and meta-analysis based on randomized controlled studies (RCTs) for exploring the advantage of EM protocol over LM protocol in respect to clinical outcomes and complications.

## Materials and methods

### Study method

Our systematic review with meta-analysis performed on the Preferred Reporting Items for Systematic Reviews and Meta-Analyses (PRISMA) guidelines (Additional file 1) [19]. Search strategy, trial selection, eligibility criteria, data collection, risk of bias assessment, and analysis process were duly conducted according to the predefined protocol (https://www.crd.york.ac.uk/PROSPERO/; PROSPERO: CRD42021240214).

### Search strategy and trial selection

The databases such as Medline, Cochrane Central Register, and Embase were searched from Jan 1, 2000, to June 30, 2021. The keywords used to explore the potential published RCTs were as follows: “Early mobilization,” “Accelerated rehabilitation,” “Delayed motion,” “Distal radius fractures,” “Distal radius,” and “Randomized Controlled Trials.” After dataset de-duplication, all titles were further filtered, and only relevant abstracts were reviewed again. Finally, the full text of eligible trials was studied before making the final inclusion. Reference lists of identified studies were also cross-checked to prevent any overlooked relevant trials.

### Inclusion criteria

Inclusion criteria were defined based on Population, Intervention, Comparison, and Outcome (PICO) method [20].

1. Population: Adults ≥ 18 years with a diagnosed DRF from acute trauma and open reduction internal fixation treatment.
2. Type of Intervention: Early mobilization group (immobilization period of ≤ 2 weeks); accelerated rehabilitation scheme (beginning of a passive and/or active wrist exercise program, immediately after internal fixation).
3. Type of Comparison: Late mobilization group (more immobilization period > 2 weeks, and then start of exercise program); standard rehabilitation scheme (No movement of the wrist until the cast removal).
4. Outcomes: At least one of the following results was required: Disabilities of the Arm, Shoulder, and Hand (DASH), Patient-Rated Wrist Evaluation (PRWE), grip strength (GS), visual analog scale (VAS), wrist range of motion (WROM) and associated complications.
5. Type of Study Design: Prospective controlled clinical trials or Randomized Controlled Trials (RCTs) published in English.

Exclusion Criteria: (1) Studies on other limb fractures other than DRF and (2) studies reporting only the radiological result.

### Data extraction

Two independent authors (KY T and HS) extracted raw data from the included studies using pre-designed data extraction tables. In the study, three or more arms were included. Then, the data were pooled from the treatment arms with the earliest motion group and the last motion group. In the studies not reporting numeric value, manual measurements of published charts were performed. Also, in the dataset, not written in standard form, the standard deviations were approximately as range/4 [21]. It is worth noting that the QuickDASH is a concept-retention version of DASH, which is similar to the complete DASH in terms of properties and scores [22]. We contacted the corresponding author to obtain the dataset, for studies containing the result of interest but with original data non-availability. Besides, any disagreements in the process were also resolved by the general consensus.

### Risk of bias assessment

The risk of bias assessment was conducted based on the Cochrane Risk of Bias Tool [23]. In addition, the quality of included RCTs was also evaluated from the following criteria: random sequence generation, allocation concealment, blinding of participants and personnel, blind outcome assessment, incomplete outcome data, selective reporting, and other sources of biases in the study. As a result, the overall quality of each study was classified as unclear, low, or high risk of bias. Meanwhile, articles with low risk of bias were also defined as four or more meeting criteria.

### Evidence assessment with the GRADE approach

The evidence assessment was performed using the guidelines of the Grading of Recommendations, Assessment, Development, and Evaluation (GRADE). The outcomes were assessed for the following elements: risk of bias, inconsistency, indirectness, imprecision, and publication bias.

### Statistical analysis

Review Manager Software (Revman 5.3.3, Cochrane Collaboration, Oxford, United Kingdom) was used for the pooled data statistical analyses. The continuous variable outcomes (DASH, PRWE, VAS, GS, WROM) were represented as mean difference (MD) with a 95% confidence interval (95% CI). Similarly, dichotomous outcomes (complications) were represented as risk ratio (RR) with 95% CI. Heterogeneity among the studies was also assessed using the I^2^ test [24], where *I*^2^ > 50% indicates significant heterogeneity and *I*^2^ < 50% was considered to have low heterogeneity; thus for the analysis, the random-effect model and fixed-effect model were used, respectively. Additionally, a *P* value of < 0.05 was considered statistically significant. Meanwhile, the MD of DASH was compared with the minimal clinically important difference (MCID), estimated at 10 in DRF to evaluate its clinical relevance [25]. The sensitivity analysis was performed to explore the reliability of the outcomes. Furthermore, publication bias was examined by Begg’s rank correlation and Egger’s weighted regression method.

## Results

### Search outcomes and trial characteristics

**Fig. 1:**
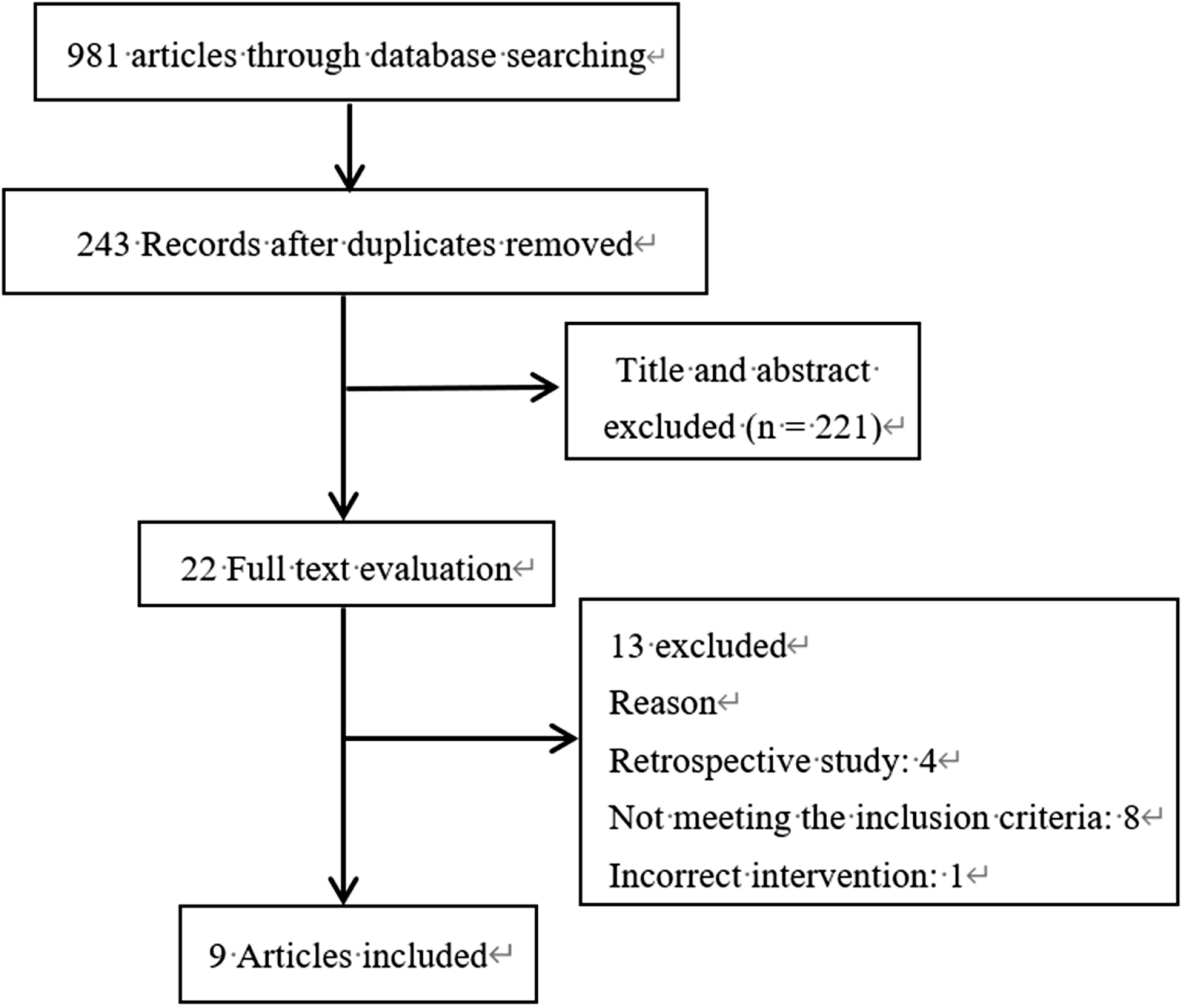
PRISMA flowchart of study selection

**Fig. 2:**
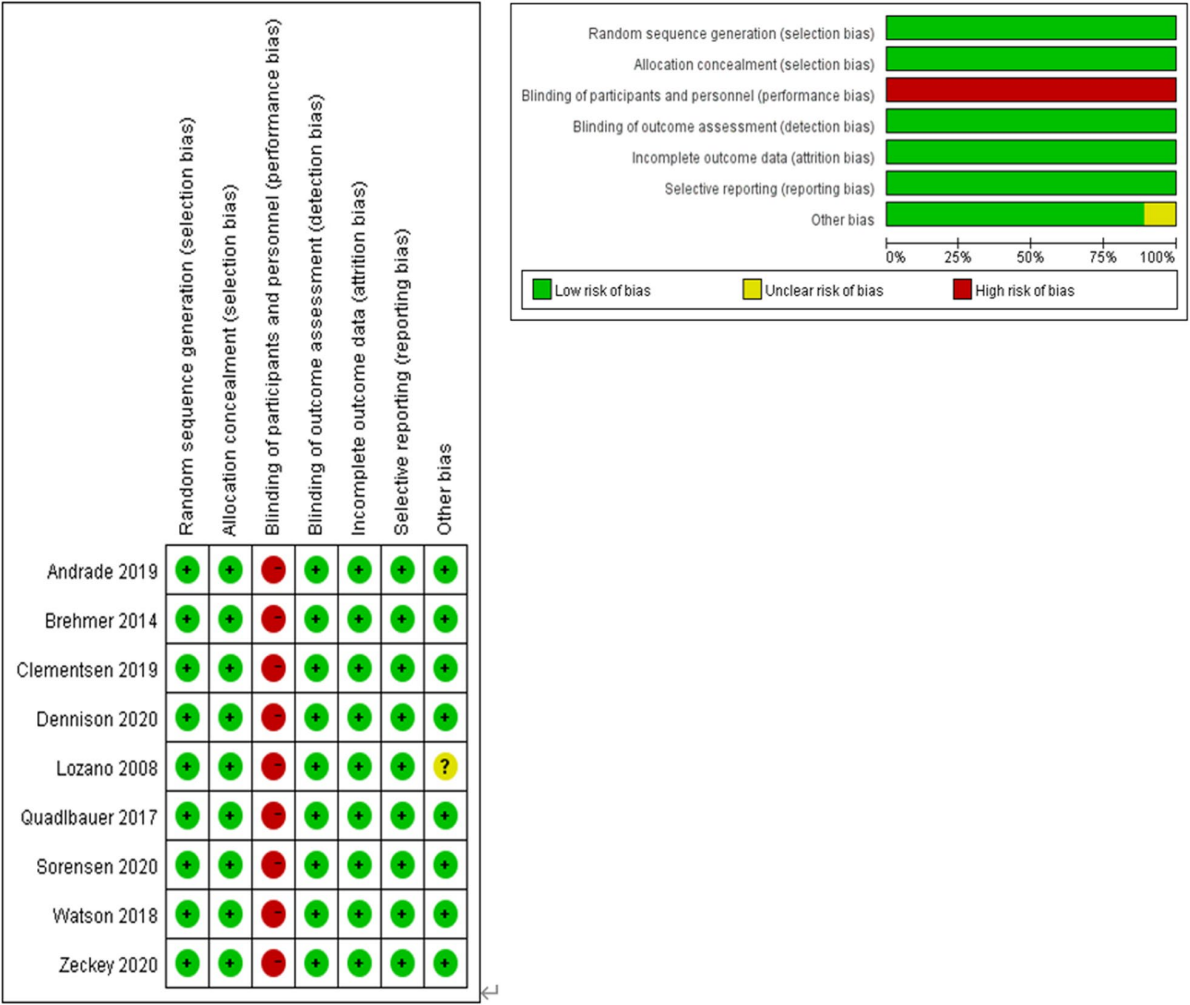
Quality assessment

As shown in Fig. 1, a total of 981 potentially relevant articles were retrieved from all the databases, but only 243 of them were retained for study, after the removal of the duplicates. Following the screening of the titles and abstracts, 221 articles were further excluded. The remaining 22 full-text articles were carefully evaluated, and 13 were excluded after the final screening due to variable reasons. Finally, 9 RCTs meeting the inclusion criteria were taken for comprehensive evaluation of this meta-analysis [10, 12–15, 17, 18, 26, 27]. Quality assessment of the included studies is shown in Fig. 2, and no investigation was excluded on bias concern. The essential characteristics of the included studies are also tabulated in Table 1. The sample sizes of the 9 studies ranged between 30 and 119. Whereas between the 293 EM and 303 LM cases, no significant differences were observed in participants demographics or fracture type.

**Table 1:** Characteristics of the included studies

References	Country	No. of patients (EM/LM)	Age(years) (EM/LM)	Male (%) (EM/LM)	AO classification (% of A:B:C) (EM/LM)	Intervention		Outcome measures
						EM	LM	
Lozano [14]	USA	30/30	55/51	37/33	40:3:57/37:23:40	Removed patients’ thermoplastic splint and performed active and active-assisted wrist motion exercises at postoperative 1st week	Wore thermoplastic splint at all times. Wrist motion exercises were initiated at 6-weeks	DASH, VAS, WROM, GS, complications
Brehmer [26]	USA	36/42	49.8/55.3	25/29	67:3:30/55:5:40	Started wrist/forearm passive range of motion and strengthened exercise at 2-weeks	Started wrist passive range of motion and strengthened exercise at 6-weeks	DASH, WROM, GS, complications
Quadlbauer [12]	Austria	15/15	49.1 ± 15.4/58.8 ± 12.1	13/15	7:7:86/0:0:100	Wore a removable thermoplastic splint on day 1 after surgery for 1 week	Wore a non-removable plaster cast for 5 weeks after surgery	DASH, PRWE, VAS, WROM, complications
Watson [17]	Australia	46/46	54.0 ± 15.6/52.0 ± 15.9	37/24.4	9:77:14/11:67:22	After cast removal at 1st week postoperatively, a standardized education and exercise program was adopted for 6 weeks	After cast removal at 6-weeks, a standardized education and exercise program was adopted for 6 weeks	DASH, PRWE, VAS, WROM, GS, complications
Andrade [15]	Brazil	19/20	51.2 ± 16.6/47.6 ± 15.1	42/45	0:0:19/0:5:95	Early wrist mobilization with inelastic bandage after surgery	A short forearm splint for 2 weeks after surgery	DASH, PRWE, VAS, WROM, GS, complications
Clementsen [13]	Norway	57/62	55 ± 12.4/55 ± 11.9	7/11	100:0:0/100:0:0	Plaster splint was removed at postoperative 2–3 days. Patients met with the institution's physiotherapist every other week during the first 3 months	Patients wore dorsal splint for 2 weeks and only met with the physiotherapist once splint was removed	DASH, PRWE, VAS, WROM, GS, complications
Sørensen [10]	Denmark	47/48	67.1 ± 8.4/67 ± 8.5	NR	36:19:45/33:10:57	Wore removable orthosis (wrist lacer) last 2 weeks, and then started nonweight-bearing exercises of fingers and wrist from the postoperative 1st day	Wore standard dorsal plaster cast for 2 weeks, and then wore removable orthosis to exercises	DASH, WROM, GS, complications
Dennison [18]	USA	18/15	54.9 ± 18.4/53.1 ± 14.6	6/7	67:33:0/67:33:0	Initiated an active and passive wrist motion protocol at postoperative 14 days	Delayed wrist motion was initiated at postoperative 5 weeks	DASH, PRWE, VAS, WROM, GS, complications
Zeckey [27]	Germany	25/25	82 ± 3/80 ± 3.5	6/8	16:4:80/8:8:84	Performing patients’ own training frequency with early mobilization and a pain-adapted increase in weight-bearing without immobilization	Wrist orthosis in a functional position for 4 weeks	WROM, GS, complications

*EM* early motion, *LM* late motion, *NR* not report, *DASH* the Disabilities of the Arm, Shoulder, and Hand, *PRWE* Patient-Rated Wrist Evaluation, *WROM* wrist range of motion, *GS* grip strength

### Primary outcome

#### DASH scores

**Fig. 3:**
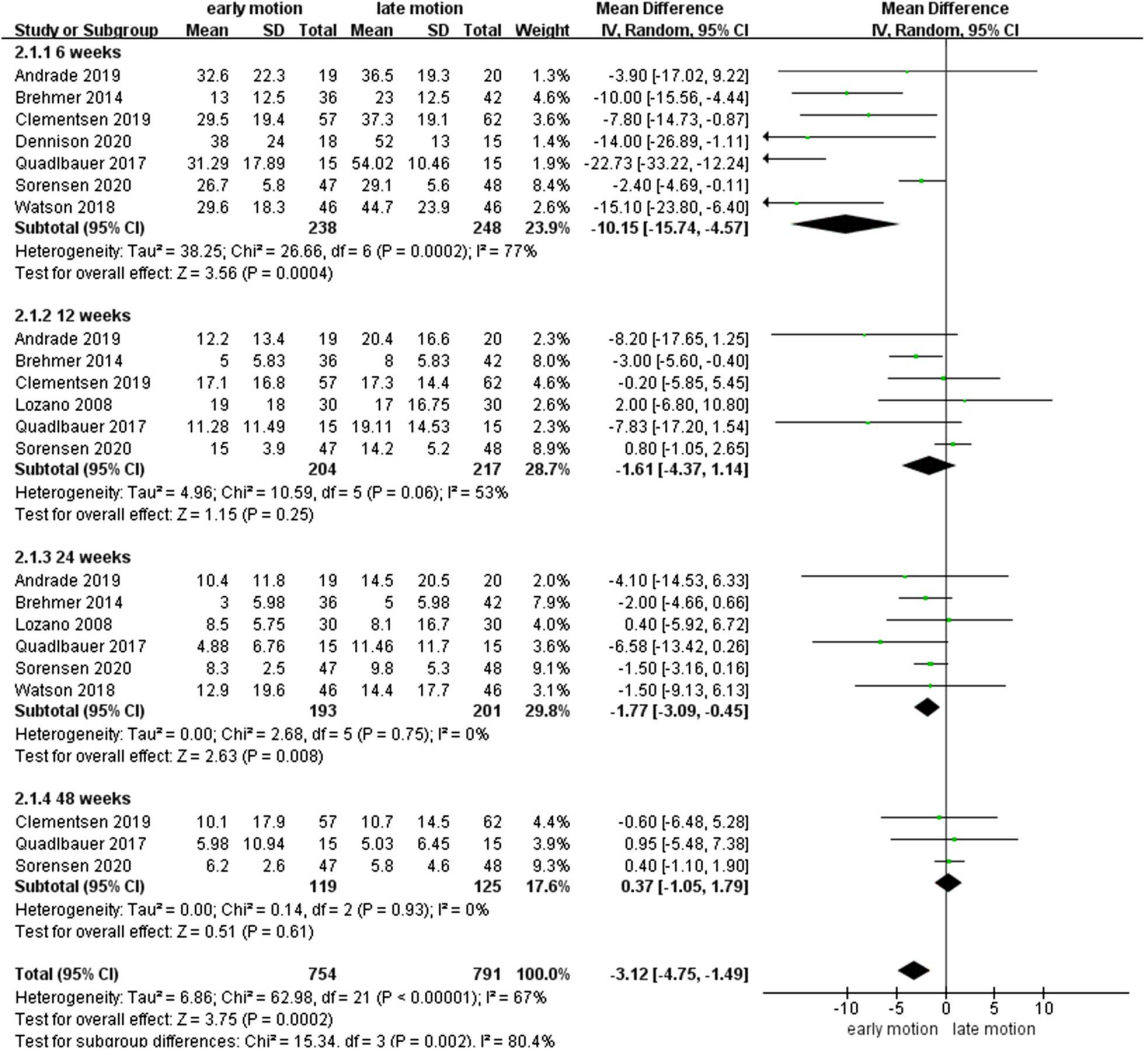
Forest plot of Disabilities of the Arm, Shoulder, and Hand scores in a meta-analysis

As shown in Fig. 3, DASH scores were available in 8 studies [10, 12–15, 17, 18, 26] for total of 546 patients. The DASH scores in EM were significantly better when compared with LM at 6 and 24 weeks postoperatively, with mean differences (MDs) of − 10.15 (95% CI − 15.74 to − 4.57, *P* < 0.01) and − 1.77 (95% CI − 3.09 to − 0.45, *P* < 0.01), respectively. Interestingly, MD at the 6th week reached the MCID value of 10. However, EM had a similar outcome to LM at the 12th and 48th week postoperatively, with MDs of − 1.61 (95% CI − 4.37–1.14, *P* = 0.25) and 0.37 (95% CI − 1.05–1.79, *P* = 0.61), respectively. The summarized outcomes were also evaluated as a moderate or lower heterogeneity, with *I*^2^ = 77%, 53%, 0%, and 0% for the 6th, 12th, 24th, and 48th week postoperatively, respectively.

### Secondary outcomes

#### PRWE scores

**Fig. 4:**
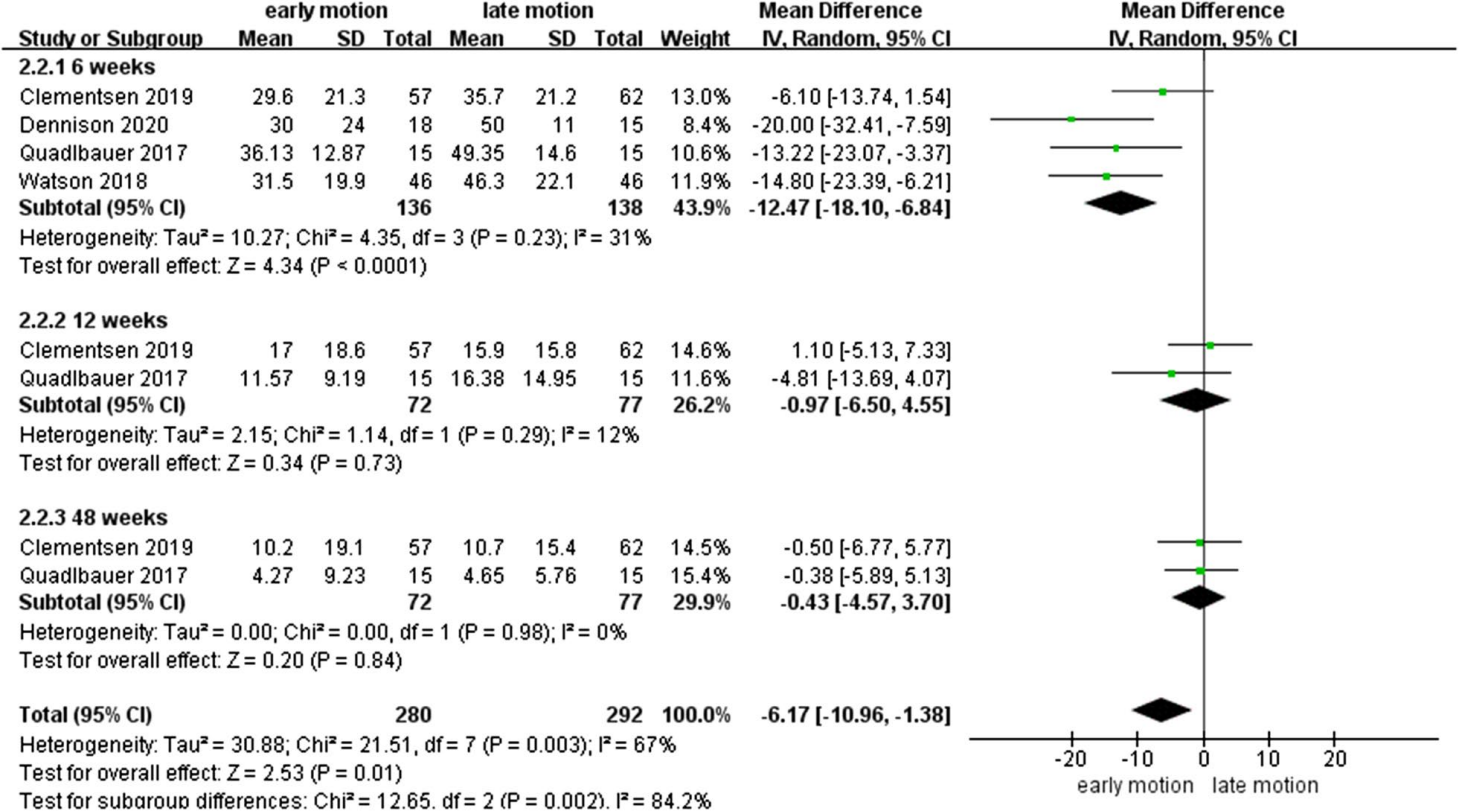
Forest plot of Patient-Rated Wrist Evaluation scores in a meta-analysis

As shown in Fig. 4, four studies [12, 13, 17, 18] with 274 patients reported data on PRWE and lower heterogeneity (*I*^2^ = 31%, *I*^2^ = 12%, *I*^2^ = 0%) for PRWE scores at 6th, 12th, and 48th week postoperatively. The outcome showed that the EM group had improved PRWE scores than the LM group, with MD of − 12.47 (95% CI − 18.10 to − 6.84, *P* < 0.01) at 6 weeks postoperatively.

### VAS scores

**Fig. 5:**
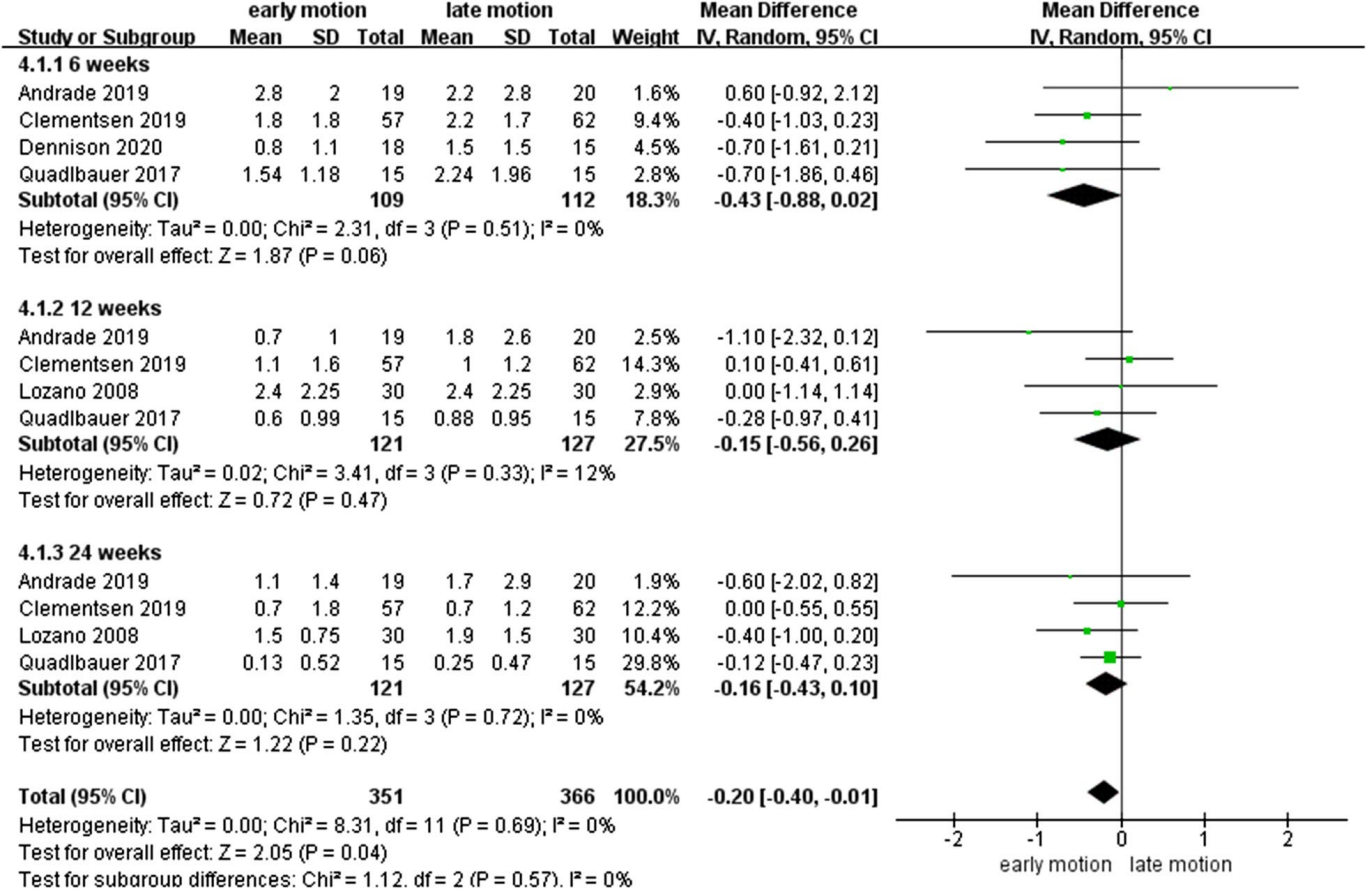
Forest plot of visual analog scale scores in a meta-analysis

As shown in Fig. 5, five studies [12–15, 18] with 281 patients had data on VAS scores. Lower heterogeneity was found for the VAS scores (*I*^2^ = 0%, *I*^2^ = 12%, *I*^2^ = 0%) at 6th, 12th, and 48th week postoperatively, although no significant differences were observed between EM and LM group (*P* > 0.05).

### GS

**Fig. 6:**
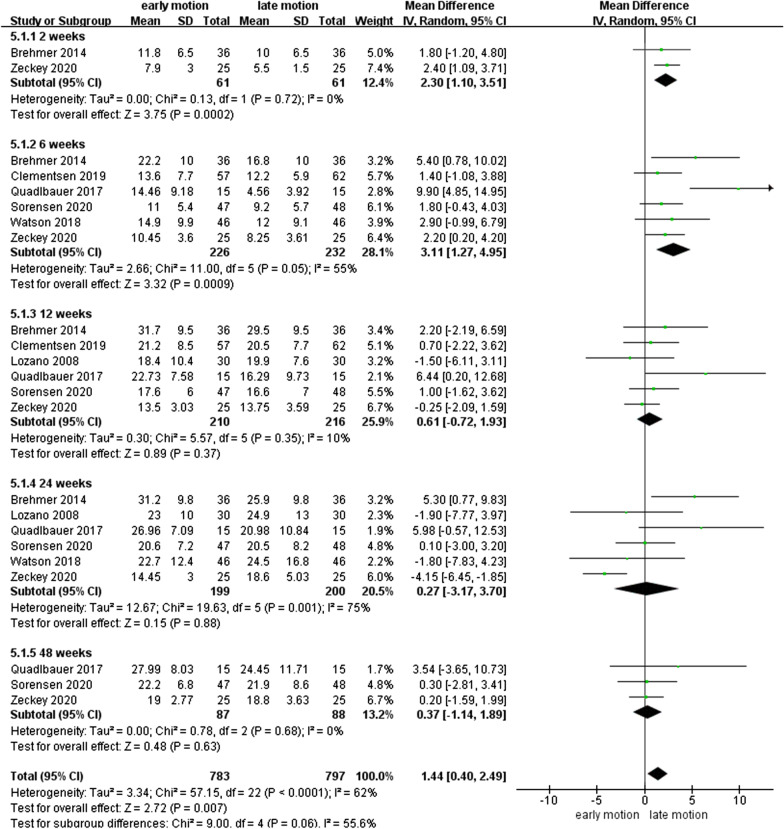
Forest plot of grip strength in a meta-analysis

As shown in Fig. 6, seven studies [10, 12–14, 17, 26, 27] with 518 patients described data on the grip strength (Kg). The summarized outcomes were evaluated as a slightly moderate or lower heterogeneity, with *I*^2^ = 0%, 55%, 10%, 75%, and 0% at the 2nd, 6th, 12th, 24th, and 48th week postoperatively. Meta-analysis showed that the EM group had a statistically better grip strength than the LM group at 2nd and 6th week postoperatively, with MD of 2.30 (95% CI 1.10–3.51, *P* < 0.01) and 3.11 (95% CI 1.27–4.95, *P* < 0.01), respectively.

### WROM

**Fig. 7:**
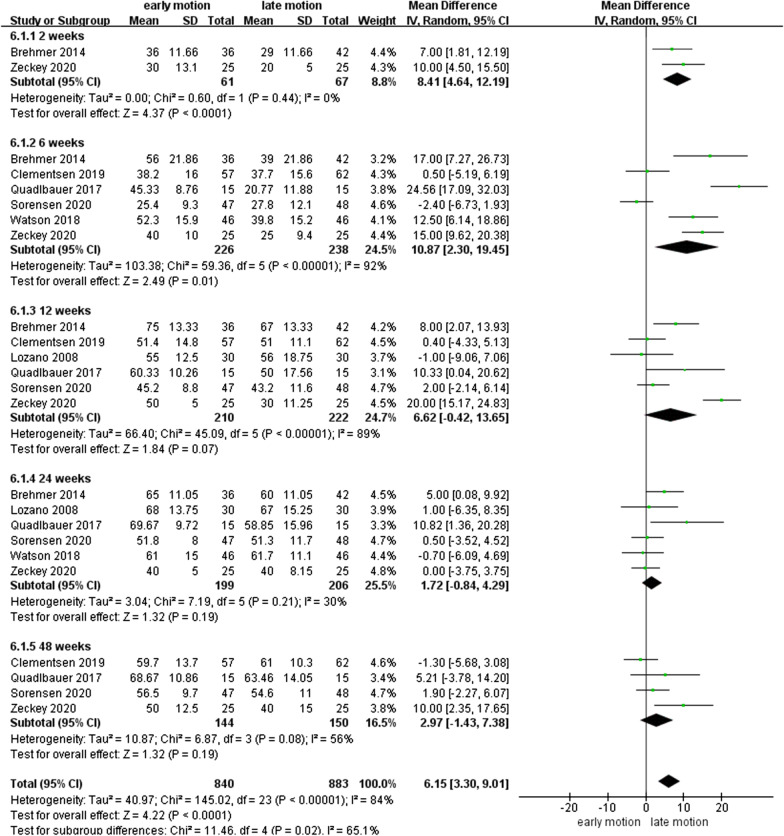
Forest plot of flexion in a meta-analysis

**Fig. 8:**
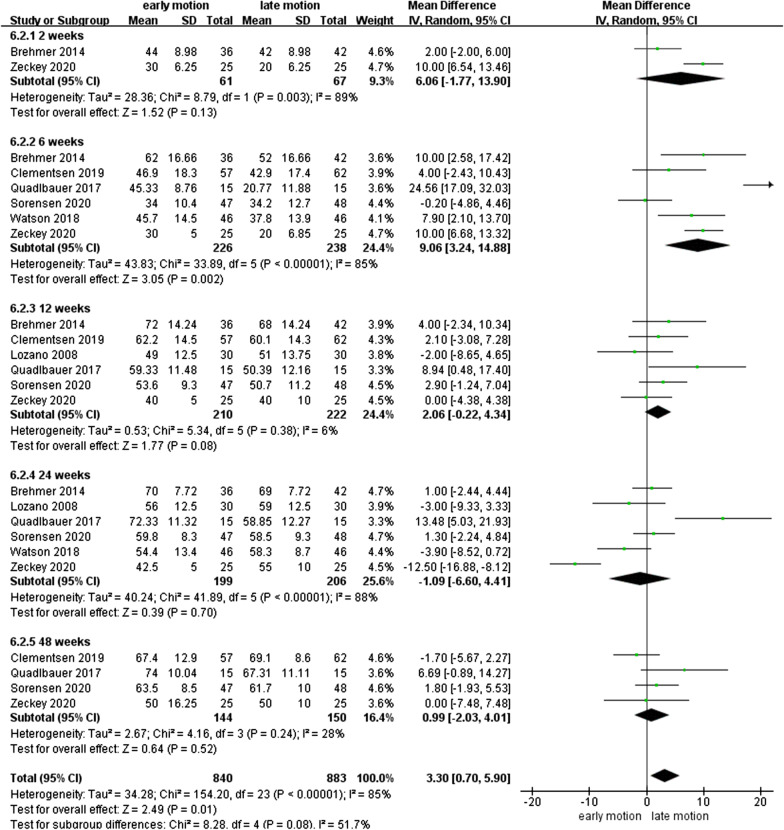
Forest plot of extension in a meta-analysis

**Fig. 9:**
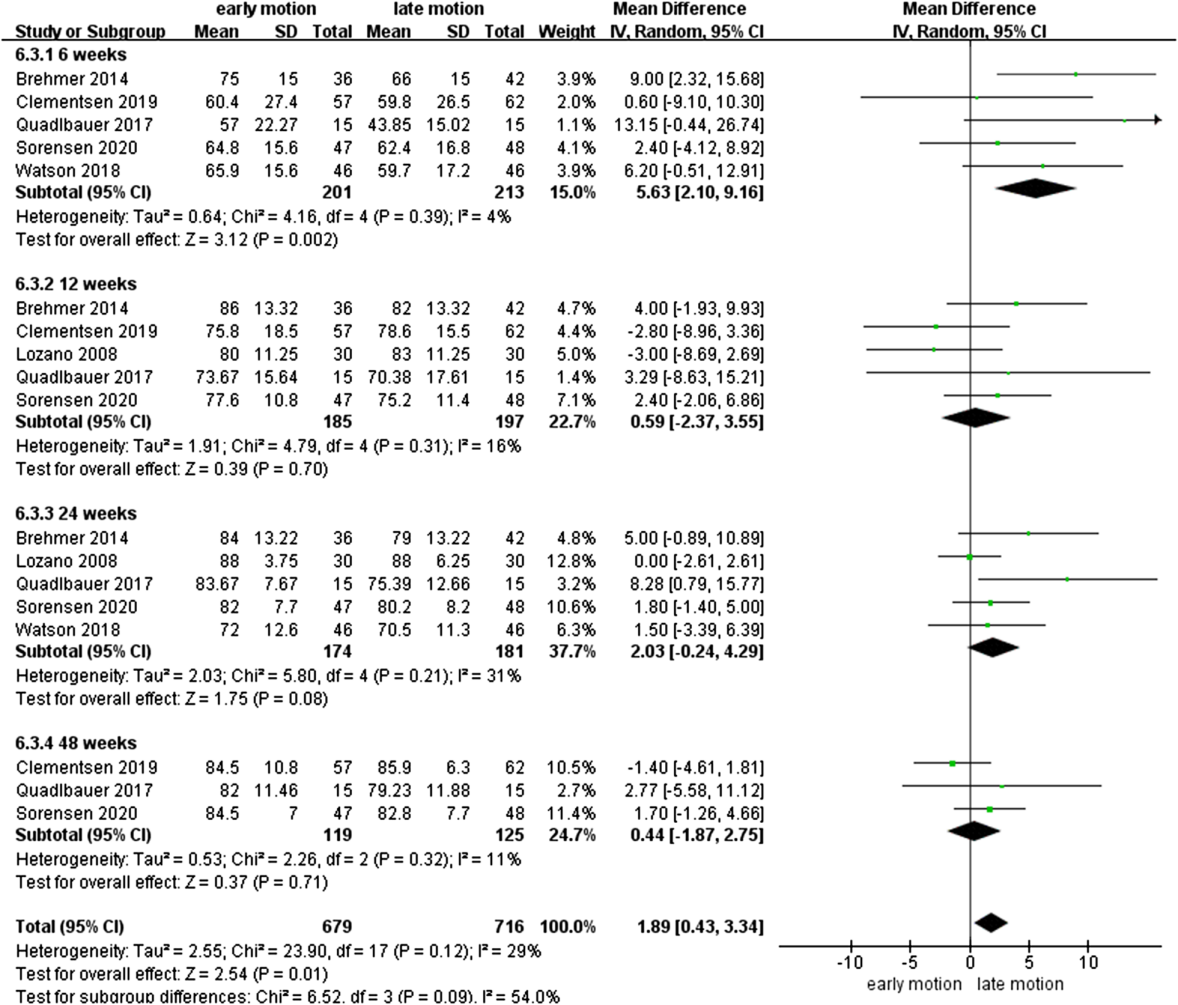
Forest plot of supination in a meta-analysis

**Fig. 10:**
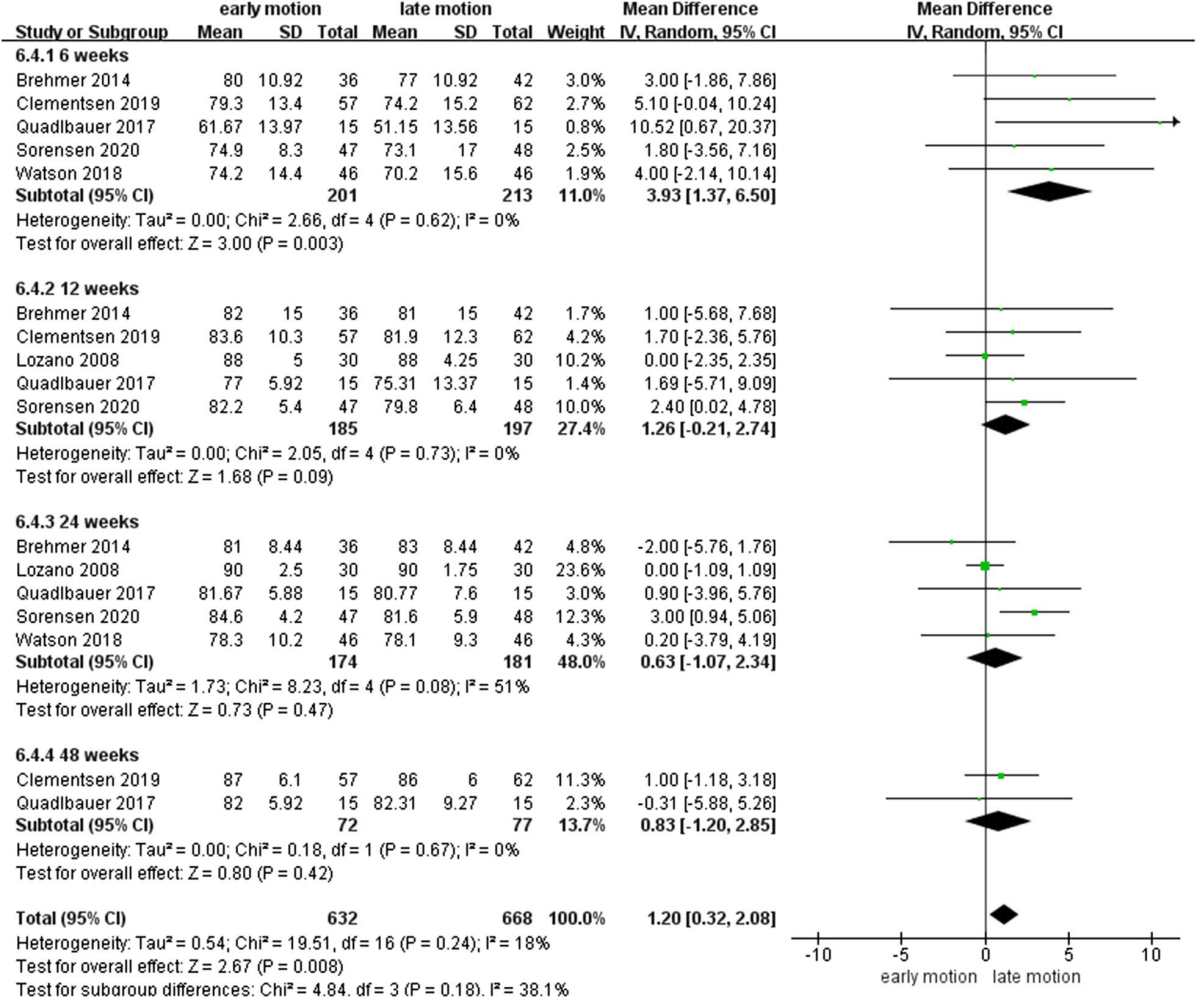
Forest plot of pronation in a meta-analysis

**Fig. 11:**
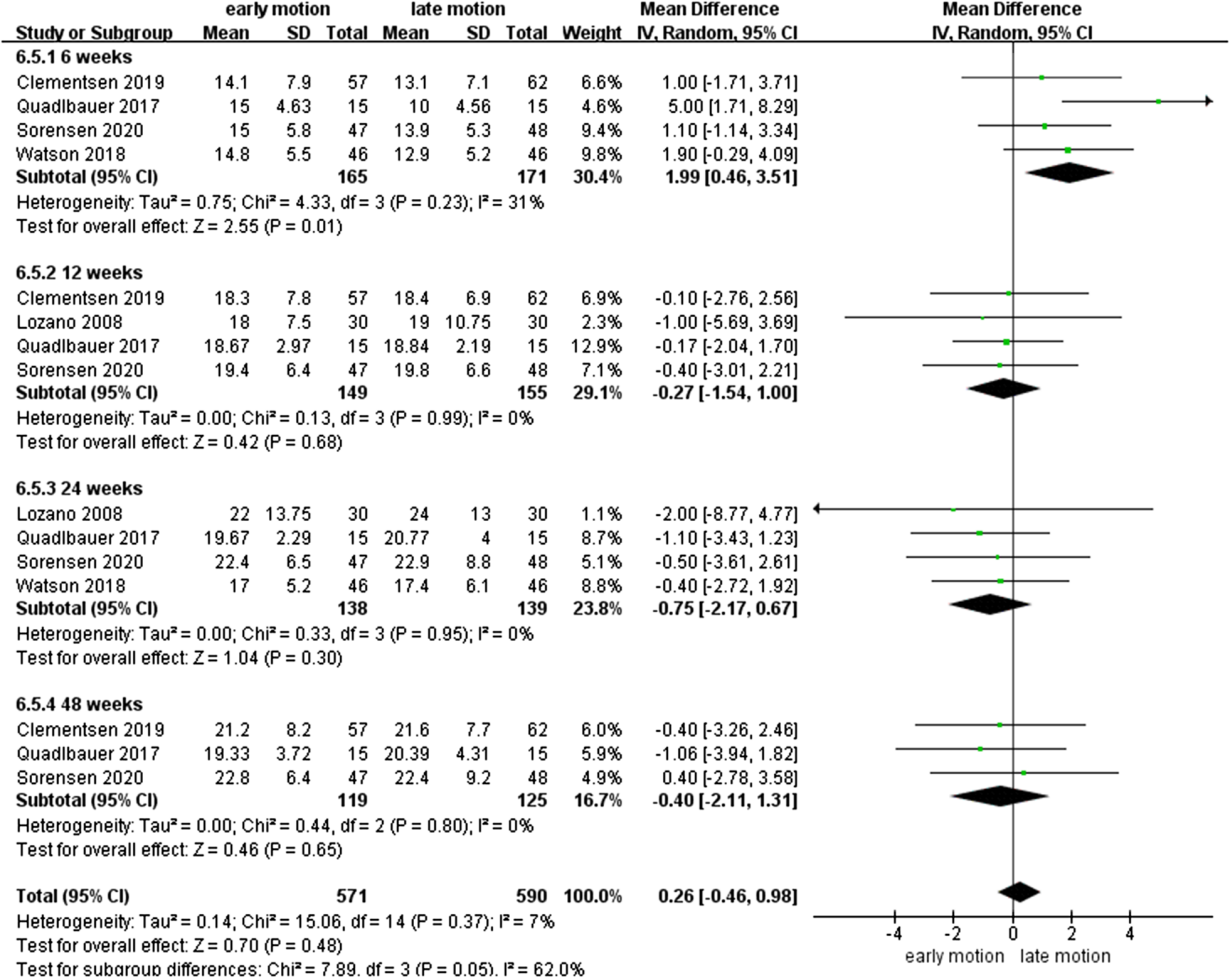
Forest plot of radial deviation in a meta-analysis

**Fig. 12:**
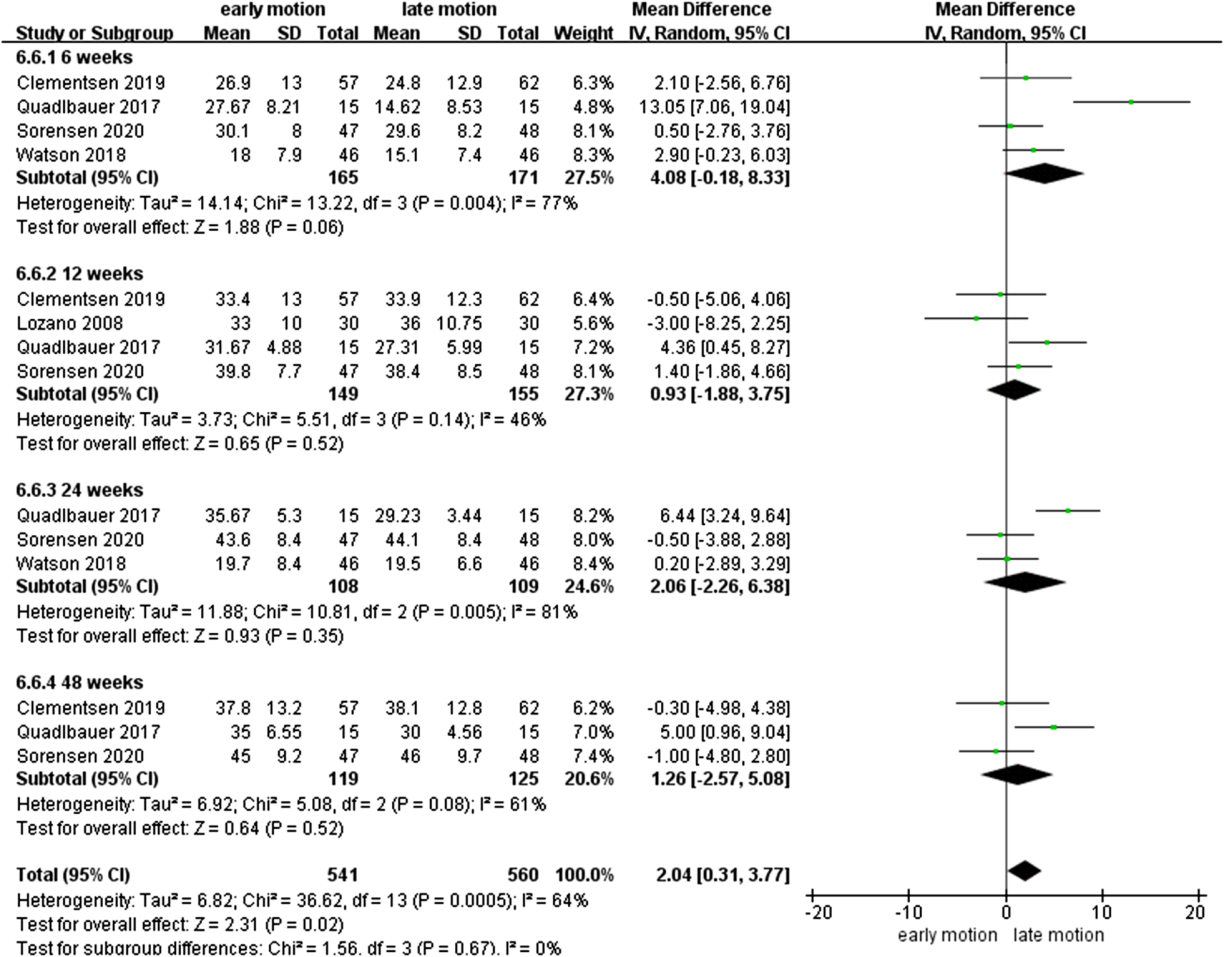
Forest plot of ulnar deviation in a meta-analysis

As shown in Figs. 7, 8, 9, 10, 11 and 12, the WROM was reported in six directions in the pooled flexion, extension, supination, pronation, radial deviation, and ulnar deviation. At the 6th week, flexion (MD = 10.87, 95% CI 2.30–19.45, *P* = 0.01), extension (MD = 9.06, 95% CI 3.24–14.88, *P* < 0.01), pronation (MD = 3.93, 95% CI 1.37–6.50, *P* < 0.01), supination (MD = 5.63, 95% CI 2.10–9.16, *P* < 0.01) and radial deviation (MD = 1.99, 95% CI 0.46–3.51, *P* = 0.01) had better performance in the EM group in comparison with the LM group.

### Complications

**Fig. 13:**
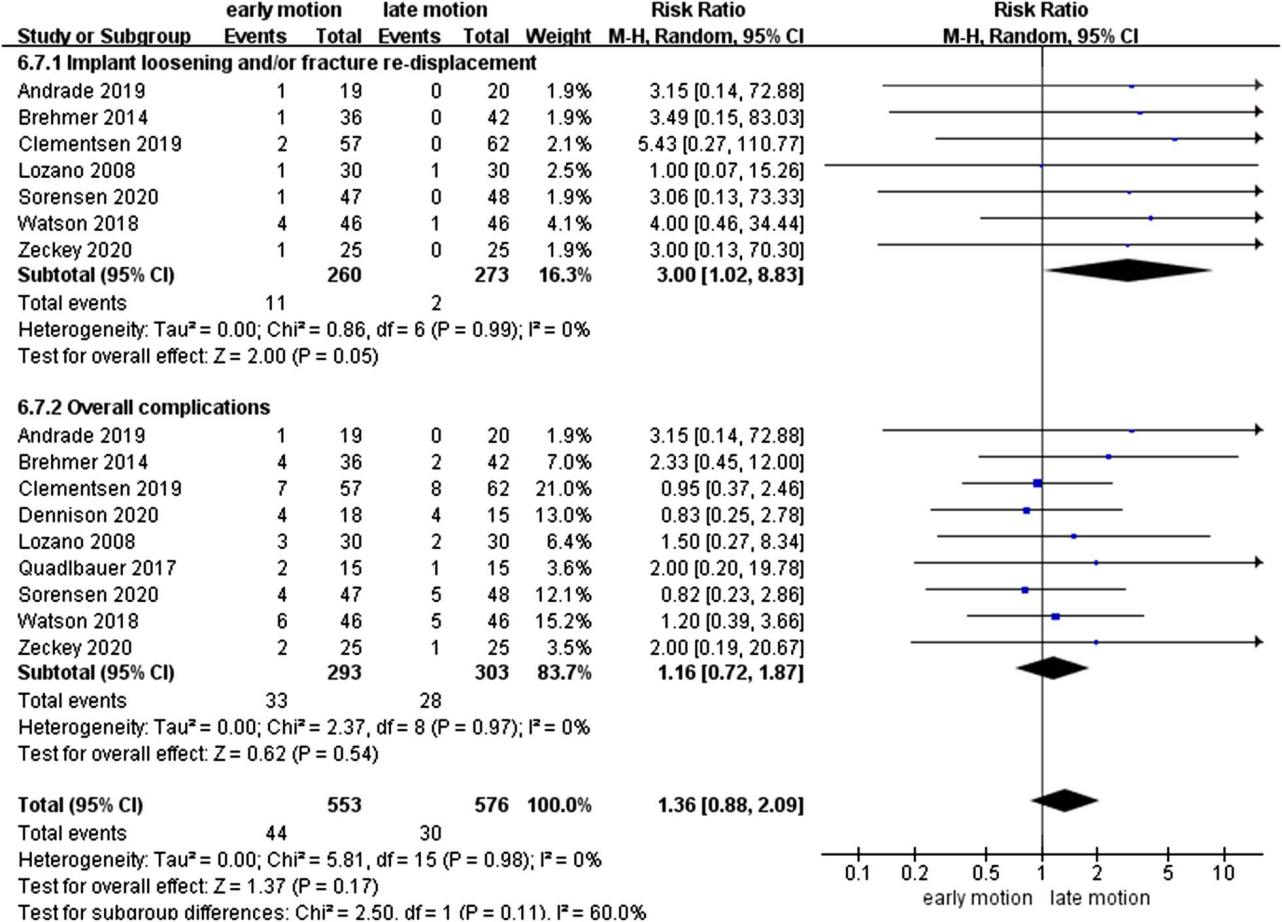
Forest plot of complications in a meta-analysis

As shown in Fig. 13, nine RCTs [10, 12–15, 17, 18, 26, 27] with 596 patients recorded related complication rates. However, no heterogeneity (*I*^2^ = 0%, *I*^2^ = 0%) from the implant loosening and/or fracture re-displacement complication and overall complications was detected. Interestingly, the pooled result on the rate of implant loosening and/or fracture re-displacement complications showed that the EM led to a potentially higher proportion than LM, with RR of 3.00 (95% CI 1.02–8.83, *P* = 0.05). The overall complications rate outcome had no statistical difference between the two groups (RR = 1.16, 95% CI 0.72–1.87, *P* = 0.54). The detailed occurrence of complications is listed in Table 2.

**Table 2:** Details of complications

Complication	No. of studies	EM	LM
Complex regional pain syndrome/prolonged pain	7	9	15
Carpal tunnel syndrome	5	10	10
Tendon rupture	5	3	1
Implant loosening and/or fracture re-displacement	7	11	2

### Heterogeneity analyses

Heterogeneity in data was resulted due to the inconsistencies found in the intervention protocol between the included RCTs. The sensitivity analysis was conducted to explore the impact of individual studies by excluding one study at each time. In the DASH, GS, flexion, extension, pronation, and ulnar deviation pooled analysis, heterogeneity showed a significant reduction when excluding one or two studies. Still, no significant difference was observed in comparison with previous results. The detailed outcomes are shown in Additional file 2.

### Publication bias

Begg's rank correlation and Egger's weighted regression analysis were performed separately to investigate the publication bias. A *P* value of < 0.05 was considered publication bias. The *P* values for all pooled analyses are presented in Additional file 3(all *P* > 0.05). No obvious publication bias was found in all the studied outcomes.

### Quality of the evidence in the GRADE system

As shown in Additional file 4, a total of 43 outcomes, including subgroup analysis of this meta-analysis, were evaluated by the GRADE system. The evidence quality for all outcomes was either moderate or low, suggesting our meta-analysis had overall moderate evidence quality.

## Discussion

The primary finding of this study revealed that EM yielded a significantly better DASH score than LM at 6 weeks (MD of − 10.15 points) postoperatively, in patients with acute displaced DRFs followed by ORIF. Moreover, this difference reached the MCID defined as 10 points in DASH [28]. Although the mean difference at 24-week DASH between EM and LM was statistically different (MD = −1.77 points, P < 0.05), which did not reach MCID (10 points). EM group also outperformed the LM group in PRWE at 6 weeks. However, the EM group had a similar clinical outcome score at 12 weeks to final follow-up (≥ 1 year) compared to the LM. The primary finding revealed that at the earlier stages, the function of injured limbs recovers more quickly in EM cohorts with DRF of ORIF.

Secondary findings at postoperative 2^nd^ and 6^th^ week showed a significantly better GS for EM compared to the LM. Nonetheless, comparing with LM, EM might be involved in a similar VAS score at 1-year follow-up. Regarding WROM, in postoperative 6^th^ week, EM showed significant improvement in terms of flexion, extension, pronation, supination, and radial deviation than LM. However, EM showed a potentially higher proportion of implant loosening and/or fracture re-displacement complications than LM (*P* = 0.05).

Postoperative EM improved the patient's quality of life and physical comfort [10, 29], and therefore assures the individuals early return to activities of daily living and work. Despite the lack of supporting studies demonstrating its effectiveness, immobilization has been empirically used to provide analgesia after surgery [30, 31]. The latest Cochrane Database Review published in 2015 by Handoll et al. [8] on rehabilitation for DRFs pointed out that as in 2006 [32], there is a lack of sufficient evidence about the effectiveness of the various rehabilitation programs after ORIF for DRF. Considering the biomechanical studies, the fixation of DRFs with a locking plate provides a five times higher stability than the forces caused by the active finger movement [33], suggesting the internal fixation treatment offers a strong fixation that meets the need for the early mobilization of these patients. However, the included studies had slight variable definitions of early mobilization. For example, Sørensen et al. [10] instructed the EM group to start nonweight-bearing exercises of the wrist and fingers from the postoperative first day, whereas Dennison et al. [18] required EM patients to gradually start active and passive wrist exercises on the 14th day after surgery. However, most of the studies included gradual movement of the wrist joints without a rigid fixation within 2 weeks or immediately after the operation.

To obtain a comparable and conclusive outcome, researches on EM versus LM after ORIF for DRFs with more high-quality large RCTs were included. The results suggested that patients treated with EM may get better clinical outcomes at an early stages which was in-concurrence with the previous studies [13, 17, 18, 26, 27]. As there was no statistical difference observed for the long term between the two groups, the difference in the functional results during the early stages between the two groups might have caused by the residual rigidity of the cast in the LM group [17]. However, at the 6th week, the pain scores of the two groups were similar, which was consistent with the study of Dennison et al. [17]. Furthermore, these results were possibly influenced by the imbalance in opioid use between the two groups. Andrade et al. [15] have shown that patients with EM tend to use more tramadol; therefore, we could not make a clear conclusion on the pain score. However, the pooled analysis showed that the EM group had a potentially higher risk of implant loosening and/or fracture re-displacement complication (*P* = 0.05), which occurred 5.5 times more (11:2) in EM than the LM group. Although the slight difference in implant loosening could be a complication resulting from immature surgical technology [10], which is still a new discovery compared to the previous literatures that compared EM with LM [13, 17, 27]. EM for patients with DRF after ORIF fracture positively affects functional recovery, but the risk of failure for fracture healing must also be considered [17], as it increases the risk of secondary surgery for these patients. The complications do have negative impact on healthcare budget as well as on the patient’s total well-being. Consequently, EM is not completely safe and flawless, so we recommend in exercise caution for extrapolating our outcomes, especially for the health care policymakers and patients.

The current study had some limitations. Firstly, our study was limited by the number of matches and available RCTs in the database; therefore, we could not perform the subgroup analysis and the pooled analysis for radiographic outcomes. Secondly, the included patients in each of the 9 RCTs were slightly different for the age bracket and mechanism of injury, as DRF in the elderly is often caused by low energy injury, while in young people, it is often associated with high energy injury and accompanied by polytrauma. Therefore, our results may vary due to variable age range. Thirdly, variation in internal fixed implants of included studies may also affect the outcomes. Fourthly, our research does not have a cost–benefit analysis as we could not remark on the potential cost differences of EM from the perspective of patients or society. Consequently, we could not remark on whether EM has the theoretical advantage of returning to work faster than LM. Lastly, each study had a different detailed rehabilitation program for the EM, which had an inherent impact on the functional scores.

## Conclusion

We showed that although EM had significantly better DASH, PRWE, GM, and WROM at earlier stages, EM and LM had similar clinical outcomes during the long-term follow-up period. Moreover, studied cases had a higher potential for implant loosening and/or fracture re-displacement complication rate when subjected to EM. Therefore, in the future, the optimal rehabilitation protocol for DRF of ORIF should be individualized, depending on the fracture types and degree of osteoporosis.

## Supplementary Information

**Additional file 1.** PRISMA checklist.

**Additional file 2.** Heterogeneity analyses.

**Additional file 3.** Publication bias of all summarized outcomes.

**Additional file 4.** Quality of evidence according to the GRADE criteria.

## Data Availability

The data sets supporting the results of this article are included within the article.

## Abbreviations

DRF: Distal radius fracture
ORIF: Open reduction internal fixation
EM: Early mobilization
LM: Late mobilization
DASH: Disabilities of the Arm, Shoulder, and Hand
PRWE: Patient-Rated Wrist Evaluation
VAS: Visual analog scale
GS: Grip strength
WROM: Wrist range of motion
MCID: Minimal clinically important difference
