# Supplementary material for: In adults, early mobilization may be beneficial for distal radius fractures treated with open reduction and internal fixation: a systematic review and meta-analysis
Source: J Orthop Surg Res. 2021 Nov 24;16:691. doi: 10.1186/s13018-021-02837-0 (PMC8611847; doi:10.1186/s13018-021-02837-0)
Supplement: Supplementary file 2 — Additional file 2. Heterogeneity analyses. [file 13018_2021_2837_MOESM2_ESM.docx]

**
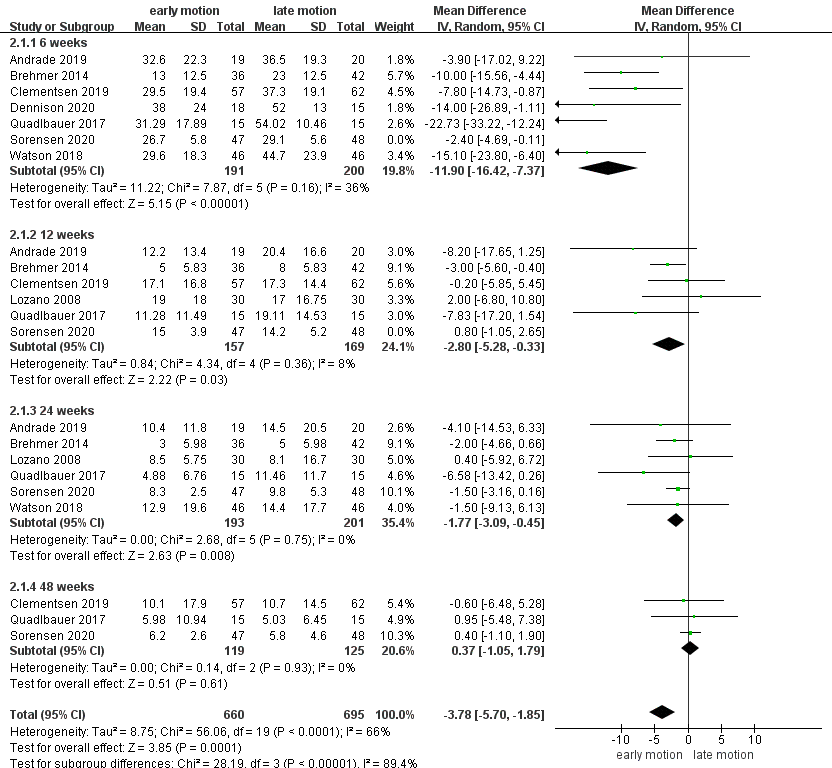
**

**Supplementary Figure 1**. Heterogeneity analysis for summarized disabilities of the arm shoulder and hand score.

**
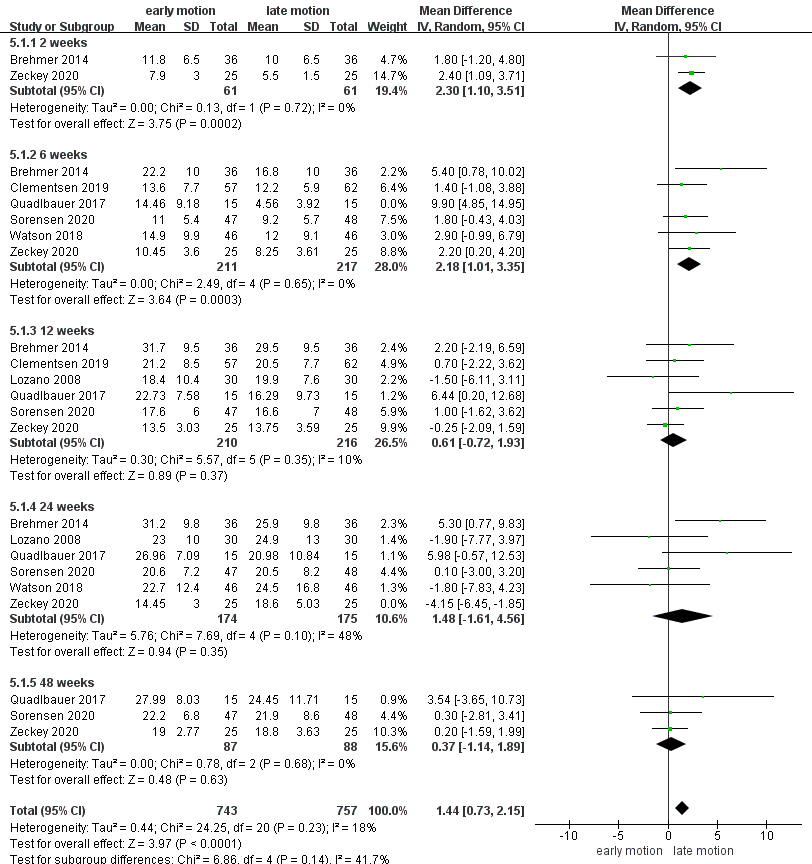
**

**Supplementary Figure 2.** Heterogeneity analysis for summarized grip strength.

**
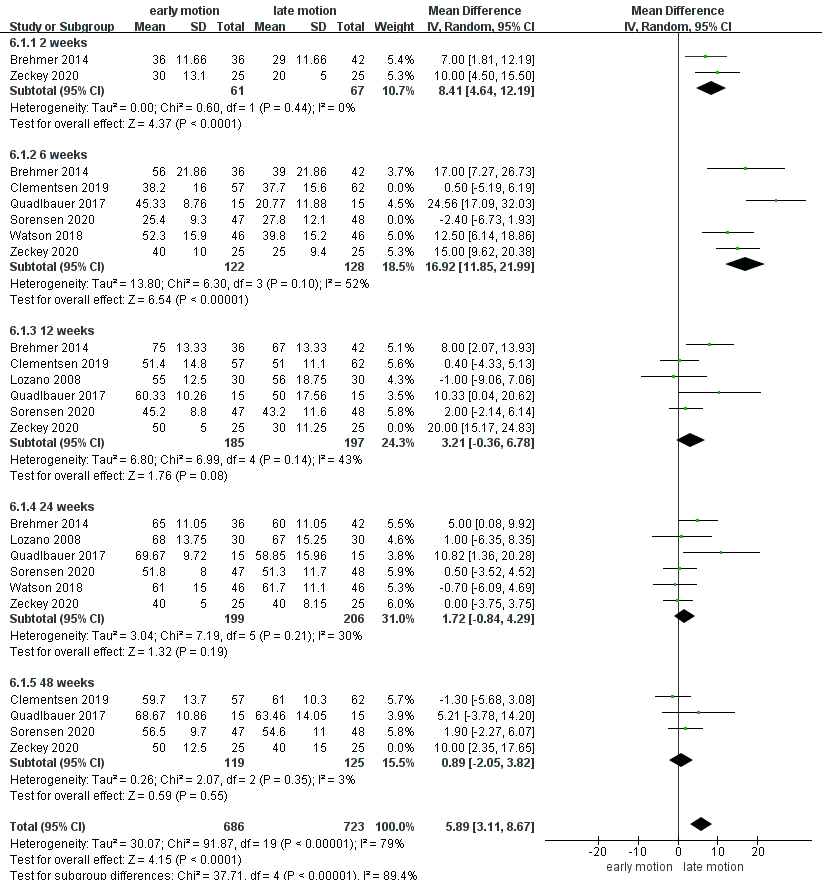
**

**Supplementary Figure 3.** Heterogeneity analysis for summarized flexion in wrist range of motion.

**
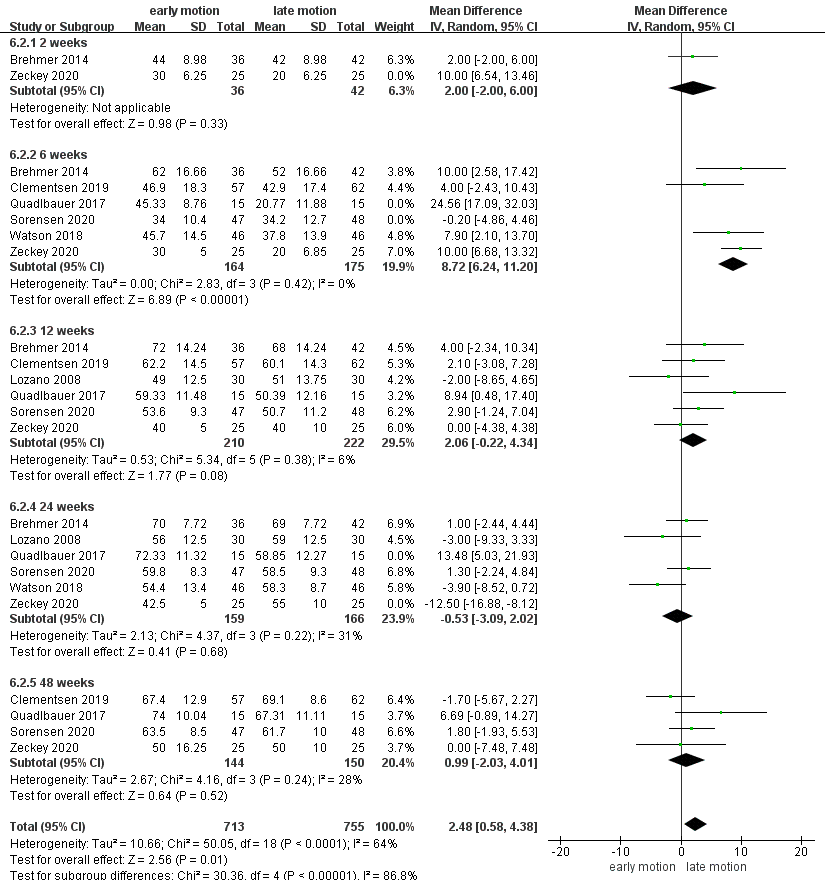
**

**Supplementary Figure 4.** Heterogeneity analysis for summarized extension in wrist range of motion.

**
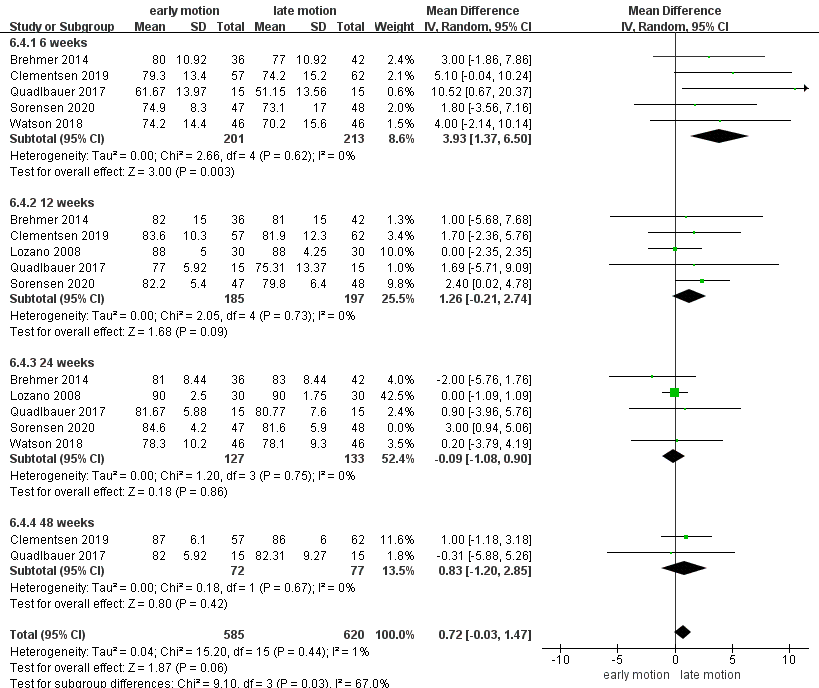
**

**Supplementary Figure 5.** Heterogeneity analysis for summarized pronation in wrist range of motion.

**
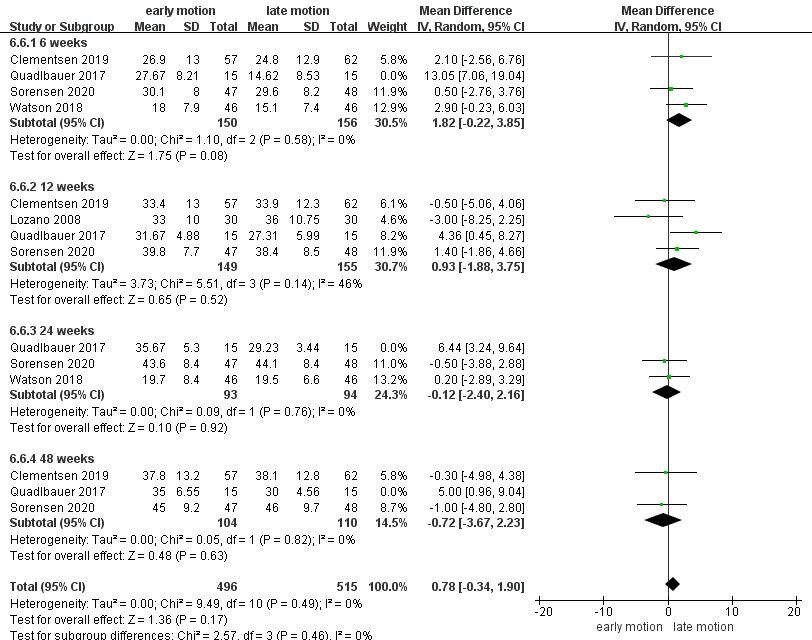
**

**Supplementary Figure 6.** Heterogeneity analysis for summarized ulnar deviation in wrist range of motion.
