# Supplementary material for: In adults, early mobilization may be beneficial for distal radius fractures treated with open reduction and internal fixation: a systematic review and meta-analysis
Source: J Orthop Surg Res. 2021 Nov 24;16:691. doi: 10.1186/s13018-021-02837-0 (PMC8611847; doi:10.1186/s13018-021-02837-0)
Supplement: Supplementary file 3 — Additional file 3. Publication bias of all summarized outcomes. [file 13018_2021_2837_MOESM3_ESM.docx]

**Supplementary 3.** Publication bias of all summarized outcomes

| **Outcomes** | **Begg (*P* value)** | **Egger (*P* value)** |
| --- | --- | --- |
| Summarized DASH after 6 weeks | 0.76 | 0.25 |
| Summarized DASH after 12 weeks | 0.45 | 0.30 |
| Summarized DASH after 24 weeks | 0.45 | 0.44 |
| Summarized DASH after 48 weeks | 0.60 | 0.82 |
| Summarized PRWE after 6 weeks | 0.31 | 0.15 |
| Summarized PRWE after 12 weeks | NA | NA |
| Summarized PRWE after 24 weeks | NA | NA |
| Summarized VAS scores after 6 weeks | 0.73 | 0.59 |
| Summarized VAS scores after 12 weeks | 0.31 | 0.29 |
| Summarized VAS scores after 24 weeks | 0.73 | 0.39 |
| Summarized grip strength after 2 weeks | 0.32 | NA |
| Summarized grip strength after 6 weeks | 0.13 | 0.40 |
| Summarized grip strength after 12 weeks | 0.26 | 0.19 |
| Summarized grip strength after 24 weeks | 0.45 | 0.17 |
| Summarized grip strength after 48 weeks | 0.30 | 0.27 |
| Summarized flexion after 2 weeks | NA | NA |
| Summarized flexion after 6 weeks | 0.26 | 0.10 |
| Summarized flexion after 12 weeks | 0.45 | 0.07 |
| Summarized flexion after 24 weeks | 0.13 | 0.19 |
| Summarized flexion after 48 weeks | 0.73 | 0.23 |
| Summarized extension after 2 weeks | NA | NA |
| Summarized extension after 6 weeks | 0.45 | 0.66 |
| Summarized extension after 12 weeks | 0.71 | 0.43 |
| Summarized extension after 24 weeks | 0.71 | 0.76 |
| Summarized extension after 48 weeks | 0.73 | 0.53 |
| Summarized pronation after 6 weeks | 0.46 | 0.11 |
| Summarized pronation after 12 weeks | 0.81 | 0.86 |
| Summarized pronation after 24 weeks | 0.46 | 0.92 |
| Summarized pronation after 48 weeks | NA | NA |
| Summarized supination after 6 weeks | 0.81 | 0.71 |
| Summarized supination after 12 weeks | 0.28 | 0.98 |
| Summarized supination after 24 weeks | 0.09 | 0.29 |
| Summarized supination after 48 weeks | 0.34 | 0.82 |
| Summarized radial deviation after 6 weeks | 0.73 | 0.27 |
| Summarized radial deviation after 12 weeks | 0.73 | 0.16 |
| Summarized radial deviation after 24 weeks | 0.73 | 0.47 |
| Summarized radial deviation after 48 weeks | 0.54 | 0.35 |
| Summarized ulnar deviation after 6 weeks | 0.73 | 0.22 |
| Summarized ulnar deviation after 12 weeks | 0.31 | 0.36 |
| Summarized ulnar deviation after 24 weeks | 0.6 | 0.89 |
| Summarized ulnar deviation after 48 weeks | 0.6 | 0.97 |
| Summarized implant loosening and/or fracture re-displacement complication | NA | NA |
| Summarized overall complications | 0.11 | 0.07 |
