# Supplementary material for: In adults, early mobilization may be beneficial for distal radius fractures treated with open reduction and internal fixation: a systematic review and meta-analysis
Source: J Orthop Surg Res. 2021 Nov 24;16:691. doi: 10.1186/s13018-021-02837-0 (PMC8611847; doi:10.1186/s13018-021-02837-0)
Supplement: Supplementary file 4 — Additional file 4. Quality of evidence according to the GRADE criteria. [file 13018_2021_2837_MOESM4_ESM.docx]

| **Supplementary 4** Quality of evidence according to the GRADE criteria | | | | | | | | | | |
| --- | --- | --- | --- | --- | --- | --- | --- | --- | --- | --- |
|  |  | No. of patients | |  | Characteristics of the included studies | | | | |  |
| Outcomes | No of studies | EM | LM | Effect size (95% CI) | Risk of bias | Inconsistency^a^ | Indirectness | Imprecision | Publication bias | Overall GRADE  quality score |
| DASH at 6 weeks | 7 | 238 | 248 | MD -10.15(-15.74, -4.57) | Not serious | Serious | Not serious | Not serious | None | ⊕⊕⊕O  Moderate |
| DASH at 12 weeks | 6 | 204 | 217 | MD -1.61(-4.37, 1.14) | Not serious | Serious | Not serious | Not serious | None | ⊕⊕⊕O  Moderate |
| DASH at 24 weeks | 6 | 193 | 201 | MD -1.77(-3.09, -0.45) | Not serious | Serious | Not serious | Not serious | None | ⊕⊕⊕O  Moderate |
| DASH at 48 weeks | 3 | 119 | 125 | MD 0.37(-1.05, 1.79) | Not serious | Serious | Not serious | Serious | None | ⊕⊕OO  LOW |
| PRWE at 6 weeks | 4 | 136 | 138 | MD -12.47(-18.10, -6.84) | Not serious | Serious | Not serious | Not serious | None | ⊕⊕⊕O  Moderate |
| PRWE at 12 weeks | 2 | 72 | 77 | MD -0.97(-6.50, 4.55) | Not serious | Serious | Not serious | Serious | None | ⊕⊕OO  LOW |
| PRWE at 48 weeks | 2 | 72 | 77 | MD -0.43(-4.57, 3.70) | Not serious | Serious | Not serious | Serious | None | ⊕⊕OO  LOW |
| VAS at 6 weeks | 4 | 109 | 112 | MD -0.43(-0.88, 0.02) | Not serious | Serious | Not serious | Not serious | None | ⊕⊕⊕O  Moderate |
| VAS at 12 weeks | 4 | 121 | 127 | MD -0.15(-0.56, 0.26) | Not serious | Serious | Not serious | Not serious | None | ⊕⊕⊕O  Moderate |
| VAS at 24 weeks | 4 | 121 | 127 | MD -0.16(-0.43, 0.10) | Not serious | Serious | Not serious | Not serious | None | ⊕⊕⊕O  Moderate |
| GS at 2 weeks | 2 | 61 | 61 | MD 2.30 (1.10, 3.51) | Not serious | Serious | Not serious | Serious | None | ⊕⊕OO  LOW |
| GS at 6 weeks | 6 | 226 | 232 | MD 3.11 (1.27, 4.95) | Not serious | Serious | Not serious | Not serious | None | ⊕⊕⊕O  Moderate |
| GS at 12 weeks | 6 | 210 | 216 | MD 0.61(-0.72, 1.93) | Not serious | Serious | Not serious | Not serious | None | ⊕⊕⊕O  Moderate |
| GS at 24 weeks | 6 | 199 | 200 | MD 0.27(-3.17, 3.70) | Not serious | Serious | Not serious | Not serious | None | ⊕⊕⊕O  Moderate |
| GS at 48 weeks | 3 | 87 | 88 | MD 0.37(-1.14, 1.89) | Not serious | Serious | Not serious | Serious | None | ⊕⊕OO  LOW |
| Flexion at 2 weeks | 2 | 61 | 67 | MD 8.41 (4.64, 12.19) | Not serious | Serious | Not serious | Serious | None | ⊕⊕OO  LOW |
| Flexion at 6 weeks | 6 | 226 | 238 | MD 10.87 (2.30, 19.45) | Not serious | Serious | Not serious | Not serious | None | ⊕⊕⊕O  Moderate |
| Flexion at 12 weeks | 6 | 210 | 222 | MD 6.62 (-0.42,13.65) | Not serious | Serious | Not serious | Not serious | None | ⊕⊕⊕O  Moderate |
| Flexion at 24 weeks | 6 | 199 | 206 | MD 1.72 (-0.84, 4.29) | Not serious | Serious | Not serious | Not serious | None | ⊕⊕⊕O  Moderate |
| Flexion at 48 weeks | 4 | 144 | 150 | MD 2.97 (-1.43, 7.38) | Not serious | Serious | Not serious | Not serious | None | ⊕⊕⊕O  Moderate |
| Extension at 2 weeks | 2 | 61 | 67 | MD 6.06 (-1.77, 13.90) | Not serious | Serious | Not serious | Serious | None | ⊕⊕OO  LOW |
| Extension at 6 weeks | 6 | 226 | 238 | MD 9.06 (3.24, 14.88) | Not serious | Serious | Not serious | Not serious | None | ⊕⊕⊕O  Moderate |
| Extension at 12 weeks | 6 | 210 | 222 | MD 2.06 (-0.22, 4.34) | Not serious | Serious | Not serious | Not serious | None | ⊕⊕⊕O  Moderate |
| Extension at 24 weeks | 6 | 199 | 206 | MD -1.09 (-6.60, 4.41) | Not serious | Serious | Not serious | Not serious | None | ⊕⊕⊕O  Moderate |
| Extension at 48 weeks | 4 | 144 | 150 | MD 0.99 (-2.03, 4.01) | Not serious | Serious | Not serious | Not serious | None | ⊕⊕⊕O  Moderate |
| Supination at 6 weeks | 5 | 201 | 213 | MD 5.63 (2.10, 9.16) | Not serious | Serious | Not serious | Not serious | None | ⊕⊕⊕O  Moderate |
| Supination at 12 weeks | 5 | 185 | 197 | MD 0.59 (-2.37, 3.55) | Not serious | Serious | Not serious | Not serious | None | ⊕⊕⊕O  Moderate |
| Supination at 24 weeks | 5 | 174 | 181 | MD 2.03 (-0.24, 4.29) | Not serious | Serious | Not serious | Not serious | None | ⊕⊕⊕O  Moderate |
| Supination at 48 weeks | 3 | 119 | 125 | MD 0.44 (-1.87, 2,75) | Not serious | Serious | Not serious | Serious | None | ⊕⊕OO  LOW |
| Pronation at 6 weeks | 5 | 201 | 213 | MD 3.93 (1.37, 6.50) | Not serious | Serious | Not serious | Not serious | None | ⊕⊕⊕O  Moderate |
| Pronation at 12 weeks | 5 | 185 | 197 | MD 1.26 (-0.21, 2.74) | Not serious | Serious | Not serious | Not serious | None | ⊕⊕⊕O  Moderate |
| Pronation at 24 weeks | 5 | 174 | 181 | MD 0.63 (-1.07, 2.34) | Not serious | Serious | Not serious | Not serious | None | ⊕⊕⊕O  Moderate |
| Pronation at 48 weeks | 2 | 72 | 77 | MD 0.83 (-1.20, 2.85) | Not serious | Serious | Not serious | Serious | None | ⊕⊕OO  LOW |
| Radial deviation at 6 weeks | 4 | 165 | 171 | MD 1.99 (0.46, 3,51) | Not serious | Serious | Not serious | Not serious | None | ⊕⊕⊕O  Moderate |
| Radial deviation at 12 weeks | 4 | 149 | 155 | MD -0.27 (-1.54, 1.00) | Not serious | Serious | Not serious | Not serious | None | ⊕⊕⊕O  Moderate |
| Radial deviation at 24 weeks | 4 | 138 | 139 | MD -0.75 (-2.17, 0.67) | Not serious | Serious | Not serious | Not serious | None | ⊕⊕⊕O  Moderate |
| Radial deviation at 48 weeks | 3 | 119 | 125 | MD -0.40 (-2.11, 1.31) | Not serious | Serious | Not serious | Serious | None | ⊕⊕OO  LOW |
| Ulnar deviation at 6 weeks | 4 | 165 | 171 | MD 4.08 (-0.18, 8.33) | Not serious | Serious | Not serious | Not serious | None | ⊕⊕⊕O  Moderate |
| Ulnar deviation at 12 weeks | 4 | 149 | 155 | MD 0.93 (-1.88, 3.75) | Not serious | Serious | Not serious | Not serious | None | ⊕⊕⊕O  Moderate |
| Ulnar deviation at 24 weeks | 3 | 108 | 109 | MD 2.06 (-2.26, 6.38) | Not serious | Serious | Not serious | Serious | None | ⊕⊕OO  LOW |
| Ulnar deviation at 48 weeks | 3 | 119 | 125 | MD 1.26 (-2.57, 5.08) | Not serious | Serious | Not serious | Serious | None | ⊕⊕OO  LOW |
| Implant loosening and/or fracture re-displacement complication | 7 | 260 | 273 | RR 3.00 (1.02, 8.83) | Not serious | Serious | Not serious | Not serious | None | ⊕⊕⊕O  Moderate |
| Over all complications | 9 | 293 | 303 | RR 1.16 (0.72, 1.87) | Not serious | Serious | Not serious | Not serious | None | ⊕⊕⊕O  Moderate |
| ^a^Studies differed in the age of participants and in the detailed post-operative interventions.  DASH: Disabilities of the arm, shoulder, and hand score; PRWE: Patient Rated Wrist Evaluation Score; VAS: Visual Analog Scale Score; GS: Grip Strength; RD: Radial deviation; UD: Ulnar deviation; MD: Mean Difference; RR: Risk Ratio. | | | | | | | | | | |
